# Emerging Roles of Exosomes in Normal and Pathological Conditions: New Insights for Diagnosis and Therapeutic Applications

**DOI:** 10.3389/fimmu.2015.00203

**Published:** 2015-05-04

**Authors:** Julieta De Toro, Leticia Herschlik, Claudia Waldner, Claudia Mongini

**Affiliations:** ^1^Centro de Estudios Farmacológicos y Botánicos (CEFyBO), Consejo Nacional de Investigaciones Científicas y Técnicas (CONICET), Universidad de Buenos Aires, Buenos Aires, Argentina

**Keywords:** exosomes, therapeutics, cancer, neurodegenerative disorders, infectious disease therapy, biomarkers, pharmacological

## Abstract

From the time when they were first described in the 1970s by the group of Johnstone and Stahl, exosomes are a target of constant research. Exosomes belong to the family of nanovesicles which are of great interest for their many functions and potential for diagnosis and therapy in multiples diseases. Exosomes originate from the intraluminal vesicles of late endosomal compartments named multivesicular bodies and the fusion of these late endosomes with the cell membrane result in the release of the vesicles into the extracellular compartment. Moreover, their generation can be induced by many factors including extracellular stimuli, such as microbial attack and other stress conditions. The primary role attributed to exosomes was the removal of unnecessary proteins from the cells. Now, several studies have demonstrated that exosomes are involved in cell–cell communication, even though their biological function is not completely clear. The participation of exosomes in cancer is the field of microvesicle research that has expanded more over the last years. Evidence proving that exosomes derived from tumor-pulsed dendritic cells, neoplastic cells, and malignant effusions are able to present antigens to T-cells, has led to numerous studies using them as cell-free cancer vaccines. Because exosomes derive from all cell types, they contain proteins, lipids, and micro RNA capable of regulating a variety of target genes. Much research is being conducted, which focuses on the employment of these vesicles as biomarkers in the diagnosis of cancer in addition to innovative biomarkers for diagnosis, prognosis, and management of cardiovascular diseases. Interesting findings indicating the role of exosomes in the pathogenesis of several diseases have encouraged researchers to consider their therapeutic potential not only in oncology but also in the treatment of autoimmune syndromes and neurodegenerative disorders such as Alzheimer’s and Parkinson’s disease, in addition to infectious diseases such as tuberculosis, diphtheria, and toxoplasmosis as well as infections caused by prions or viruses such as HIV. The aim of this review is to disclose the emerging roles of exosomes in normal and pathological conditions and to discuss their potential therapeutic applications.

In recent years, the study of extracellular vesicles (EVs) has mainly focused in a type of vesicles secreted into the extracellular compartment that were termed exosomes by Johnstone, who isolated them from sheep reticulocytes and described their endocytic origin (1). Later, other authors have described this type of vesicles and their secretory pathway. These findings have been extensively described in many comprehensive reviews (2–7).

The interest in EV and, in particular, in exosomes is reflected in many reports published earlier and the constitution of the International Society for Extracellular Vesicles in 2011 to discuss important findings in the field and a database named Exocarta (http://www.exocarta.org), created in 2009 as a free web-based resource to compile proteins and RNA identified in exosomes (8).

Extracellular vesicles are constitutively released from many cell types including neurons, tumor and immune cells, among others. They can also be found in different body fluids such as serum, breast milk, saliva (9), cerebrospinal fluid (10), semen (11), and urine (12).

Eukaryotic cells release vesicles into the extracellular environment either by direct membrane budding (ectosomes or microparticles: apoptotic vesicles, membrane particles and exosome-like vesicles) or by fusion of internal multivesicular compartments (exosomes) (7).

Exosomes are small particles of 30–100 nm with a membrane that is rich in lipids such as sphingolipids, ceramide, and cholesterol with a density between 1.15 and 1.19 g/ml (3).

Exosomes have an endocytic origin and their development begins when early endosomes, loaded with ubiquitinilated proteins, are recognized by the endosomal sorting complex required for transport (ESCRT). This recognition allows the formation of intraluminal vesicles which, in turn, give rise to the multivesicular bodies (MVBs) containing proteins. This process continues with the fusion of the MVBs with the plasma membrane releasing their content to the extracellular space; the microvesicles released are exosomes (3, 6, 13, 14). However, an independent ESCRT pathway for exosomal biogenesis and release has recently been described (6).

When exosomes are secreted outside the cells, they can follow one of these three pathways: (1) they can be captured by neighboring cells (or by the same cells that have given rise to them); (2) they can be internalized by cells that are within a certain distance; or alternatively, (3) they can enter the systemic circulation and then be taken up by different tissues (15).

Canonical exosomes can be described by the presence of molecules which are specifically associated to them, regardless of the cell type they derive from (16). For example, exosomes express some typical cytosolic proteins such as tubulin and actin, molecules involved in MVB biogenesis as TSG101 and Alix, proteins that participate in signal transduction such as protein kinase, metabolic enzymes, heat shock proteins (HSP 70, HSP 90), Annexin and Rab family proteins, tetraspanins (CD9, CD63, CD81), various transmembrane proteins, and major histocompatibility complex class I (MHC I) molecules. Particular expression of some proteins may be present, such as major histocompatibility complex class II (MHC II) on exosomes derived from antigen-presenting cells such as B-cells or dendritic cells (DCs) or some tumor antigens such as MelanA/Mart-1 (16, 17). Because of their endosomal origin, nuclear, mithochondrial, or endoplastic proteins have not been found in exosomes (13).

In addition to this protein content, exosomes bear molecules such as lipids, mRNA, and non-coding RNAs, including micro RNAs (miRNAs) that are delivered and properly translated in target cells (6, 17). The latter is an important feature because the presence of mRNA in exosomes results in the transfer of genetic information allowing protein expression to take place at a distance.

Overall, all these features have allowed characterization and isolation of exosomes from other microvesicles secreted by cells into the extracellular compartment. The classic method to isolate exosomes, is the differential centrifugation and ultracentrifugation. However, over the last few years, new methods have been developed either to obtain large quantities of exosomes to be used in clinical trials which include, for example, the ultrafiltration (18, 19) or in an attempt to obtain pure exosomes preparations, as the use of monoclonal antibodies directed to marker proteins on the surface of exosomes bound to magnetic beads (20). In addition, microvesicles have been differentiated and classified according to various characteristics such as morphology, density, expression of marker proteins, size and a very important feature: their intracellular origin.

Until recently, it has generally been accepted that EV differ in their physiochemical characteristics such as size, density as determined by their sedimentation equilibration in sucrose gradient, and the expression of main protein markers. Most of the works that have been done in the field relay on these properties to develop methods as those mentioned earlier to isolate and characterize the different EV. However, recent works have reported overlapping characteristics and functions for the EV. In fact, a single cell can produce different EV, including exosomes. In this sense, there is a general concern among scientists working in the field regarding the representativeness of the current literature data. The International Society for Extracellular Vesicles has published a paper proposing standardized methods to isolate EV. Thus, new methods and more accurate markers are under evaluation (21). Detailed evidences of the heterogeneity of EV preparations have recently been reviewed by Colombo et al. (7).

Although the primary role attributed to exosomes has been the removal of unnecessary proteins from the cells, their principal function described is their role in cell–cell communication (17). In addition, they participate in several different functions and in a large variety of pathways as they are biologically active vesicles secreted to the extracellular environment (13).

## The Role of Exosomes in Immune Function

The role of exosomes in the stimulation of the immune system has been extensively studied and a recent study in deep review has been published discussing how exosomes regulates immune response (6). In this review we will discuss some of the most relevant findings.

The original work carried out by Raposo et al. demonstrating the importance of exosomes derived from B-cells in antigen presentation and T-cell stimulation has changed the idea that these microvesicles serve merely as a clearance system for obsolete proteins (22). Since the publication of this work, numerous studies have confirmed that exosomes derived from professional antigen presenting cells, such as DCs, express class I, class II MHC, adhesion, and co-stimulatory molecules. These characteristics enable these exosomes to activate directly CD8+ and CD4+ T-cells inducing a strong immunogenic response (2, 4, 23). DCs pulsed with tumor peptides release immunogenic exosomes and elicit a strong CD8+ T-cell-dependent anti-tumor immune response (2). In fact, it has been demonstrated that exosomes can activate T-cells either by a direct antigen presentation or by an indirect presentation through transfer of antigenic peptides to APCs (24–26).

Tumor-derived exosomes express tumor antigens that can activate DCs, thereby priming the immune system to recognize and promote a specific cytotoxic response with a higher immunogenicity than that accomplished by tumor cell lysates or soluble antigens when used as vaccines (24). Moreover, a single intraperitoneal injection of tumor peptide-loaded DCs-derived exosomes can induce a very strong immune response leading to a delay in tumor growth or to a complete tumor rejection. This phenomenon is probably because of the high tumor antigen density and also of the presence of HSP with an adjuvant capacity in the microvesicles, as observed for exosomes derived from melanoma cells (24, 27–30). In this regard, Lancaster and Febbraio have determined that exosomes expressing HSP70 can activate *natural killer cells* (NKs) and macrophages (31).This rational approach has been probed in several pre-clinical studies and in Phase I (32, 33) and Phase II clinical trials that are already under investigation in patients with inoperable non-small cell lung cancer.

Other immune cells release microvesicles with immune functions, for example, NKs-derived exosomes enclose perforin and granzyme B and mediate anti-tumor activities either *in vitro* or *in vivo* (34). Furthermore, peptides expressed in exosomes released by mast cells are presented by DCs and induce specific immune responses *in vivo* (35). It has also been reported that macrophages release IL-1β on inflammasome activation, suggesting a role of these microvesicles in the pro-inflammatory activity and the innate immune response (36).

Exosomes have also been shown to induce immune suppression. Over the last years many authors have reported the immunosuppressive properties of tumor exosomes which may explain the low immunogenicity observed in some studies. For example, tumor exosomes can suppress NK cells by modulating the expression of the NKG2D receptor (37). These exosomes can promote the generation of regulatory T-cells (38); induce T-cells apoptosis through the activation of Fas (39) or tumor necrosis factor ligands (40) or by the expression of galectin 9 (41) and affect the maturation of DCs (42). The immunosuppressive properties of exosomes derived from IL-10 treated DCs or DCs genetically modified to express IL-4 have also been demonstrated in a collagen-induced arthritis model (43, 44).

Moreover, it has been demonstrated that exosomes derived from infected cells also display pathogenic antigens that can induce a specific anti-microbial immune response. For example exosomes released by endothelial cells infected with Cytomegalovirus (45)or *Mycobacterium bovis*-infected macrophages (46) can induce a specific immune response. Besides, DCs pulsed with *Toxoplasma gondii* promote anti-parasite immunity in mice (47).

## Exosomes in Cancer Disease

It is known that a direct interaction between tumor cells and their environment is essentially required for cancer progression. To achieve this cell–cell communication, an efficient information exchange must exist, being exosomes of paramount importance to induce a pro-tumoral microenvironment for carcinogenesis and regulating the immune response to promote tumor progression and survival. To accomplish these tasks, exosomes are involved in multiple mechanisms: exosomes released into the extracellular milieu participate in the remodeling of the extracellular matrix and promote angiogenesis, thrombosis, and tumor cell proliferation (48, 49).

Owing to their stability, the specific tissue uptake and their ability to transfer micro or mRNA and proteins to recipient cells, exosomes may travel to distant sites and promote a pro-tumor environment to harbor metastatic niches (50). As mentioned earlier, exosomes may additionally exert an immune suppression profile, thus favoring a tumor escape mechanism to evade the immune attack.

It has been demonstrated that exosomes can modulate nearby or distant target cells by direct contact of their surface molecules to activate intracellular pathways. Alternatively, on internalization by membrane fusion or endocytosis, exosomes deliver their protein or RNA content. Many proteins such as mutant KRAS and MET oncoprotein have been found to be transported by exosomes and that their uptake was favored by the hypoxic tumor microenvironment. Recently, studies have demonstrated that tumor cells contain different levels of miRNA and also onco-miRNA which can post-transcriptionally modulate the acceptor cell function (51). Exosomes obtained from normal prostate epithelial cells with normal levels of miRNA-16, 143, and 205 could inhibit the *in vitro* proliferation of a prostate cell line with lower concentrations of these miRNA (52).

Cytokines and soluble mediators in combination with tumor-derived exosomes can recruit bone marrow-derived cells to tumor pre-metastatic tissue where they contribute to modulate the permissive microenvironment for tumor establishment. Recently, Peinado et al. have demonstrated that melanoma exosomes containing MET oncoprotein modulate bone marrow-derived cells to generate a pro-vascular phenotype in the lung (53).

As the result of the many cellular functions in tumor development and dissemination where exosomes take place, these microvesicles represent novel biomarkers for a non-invasive and more accurate diagnosis and prognosis of the disease progression.

Exosomes have several properties that make them preferable over EV for the purpose of therapeutics, including that exosomes are stable *in vivo* and *in vitro*, bioavailable, well distributed in the organism, cross the blood–brain barrier, are well tolerate and may regulate gene expression by transferring miRNA and siRNA to target cells. Overall, these characteristics highlight the importance not only as ideal vaccines for cancer treatment, as initially considered, but also more important as natural liposomes for the delivery of biologics, allowing multiple opportunities for developing new alternatives for cancer treatment.

## Exosomes Associated to the Generation and Progression of Neurodegenerative Diseases

Exosomes have been proposed to be novel actors during normal development and physiology of the nervous system, acting as cell–cell communicators and playing functional roles not only during the development but also during the regeneration of normal neurons. Lachenal et al. have elucidated the role of exosomes in the normal physiology of the central nervous system by demonstrating the secretion of exosomes in culture by completely differentiated cortical and hippocampal neurons, being this secretion regulated by the calcium influx and by the glutamatergic synaptic activity (54). Furthermore, Frühbeis et al. have reported a reciprocal cell–cell communication between neurons and oligodendrocytes mediated by exosomes (55). Oligodendrocytes release exosomes in response to neuronal stress signals and are internalized by neurons via an endocytic pathway to deliver protective proteins, glycolytic enzymes, mRNA, and miRNA to axons, exerting neuroprotection (56).

Apart from the function of exosomes in the normal development and physiology of the nervous system, the generation and progression of many neurodegenerative diseases have been associated with exosome-mediated transport of misfolded proteins. Moreover, exosomes have been termed The *Trojan horses of Neurodegeneration* because of their capability of shipping toxic agents from unhealthy neurons to their own neighboring cells (57). This nickname has then, and not entirely correctly, associated exosomes to pathological transport mechanisms involved in several kinds of pathways that finally end in neurodegeneration.

In Parkinson’s disease, for example, α-synuclein mutated proteins form intracellular oligomers (known as Lewy’s bodies) can be secreted via exosomes to the extracellular milieu and internalized by nearby cells, thus spreading the disease from cell–cell within the brain. In this regard, Danzer et al. have characterized autophagy as a protective mechanism in cells, considering it the major degradation pathway for α-synuclein oligomers (58). Any deregulation in neuronal autophagy might promote the aggregation of these proteins and their secretion by exosome release, thus spreading the toxic seed and causing neurodegeneration. Mittelbrunn et al. have proposed that promoting autophagy (i.e., preventing cells from exosomal release) may become a novel approach in the treatment of neurodegenerative diseases (59).

Several hallmark protein accumulations have been characterized in other neurodegenerative diseases like Alzheimer’s, in which *tau* protein aggregates form filamentous intracellular inclusions that can spread from affected nerve and glial cells to healthy ones thus functioning also as potential *seeds* of the disease. Bolmont et al. and Götz et al. have shown that extracellular α-amyloid aggregates can induce *tau* pathology in transgenic mice and finally promote neurodegeneration (60, 61). Moreover, exosome-associated *tau* and α-amyloid have been described and it has been proposed that the exosomal surface can act as the seed responsible of the β-amyloid aggregation after protein conformational modifications (62).

In this regard, β-site APP-cleaving enzyme 1 (BACE 1) is responsible for the formation of the aggregates as mentioned earlier. Alvarez-Erviti et al. have accomplished the encapsulation of BACE 1 siRNA in exosomes, observing after their administration to a murine model for Alzheimer’s, a significant decrease in BACE 1 mRNA and β-amyloid levels (63). They have also demonstrated that exosomes which have the ability to cross the blood–brain barrier are non-toxic and that they can be perfectly well tolerated. These findings imply that the expression of BACE1 levels, controlled by exosome-mediated siRNA delivery, may be an interesting breakthrough in the treatment of Alzheimer’s disease.

Exosomes were also implicated in the propagation of PrPSc (the infectious agent associated with prion diseases such as Creutzfeldt–Jakob disease and Gerstmann–Sträussler–Scheinker syndrome) (64–66).

In an interesting study it was demonstrated a differential expression of specific exosomal miRNA in post-mortem brain samples in patients with Scizophrenia and bipolar disorders (miR-497 and miR-29c) compared with those from normal control samples (67).

Owing to the fact that a variety of aggregating proteins involved in neurodegenerative diseases have a direct association with exosomes, these nanovesicles have become an interesting biomarker for diagnostic and prognostic. Moreover, their capability to accomplish targeted neuron-to-neuron transport transforms them in potentially specialized carriers of therapeutic drugs for neurologic and psychiatric disorders. In addition, exosomes might be used for controlling the disease by silencing or restoring the normal content of miRNA.

## The Role of Exosomes in Cardiovascular Diseases

Cardiovascular diseases are one of the leading causes of death worldwide. Although patients can control some of the behavioral risk factors, physiological factors responsible for heart damage are major goals to be attained.

Several authors have found a direct association between cardiovascular diseases and high concentrations of circulating microvesicles. Exosomes have pro-angiogenesis, pro-coagulant and pro- and/or anti-inflammatory effects as well as an opposing impact on the vascular tone and vessel wall. These features of exosomes are probably because of their capability of transporting and cell–cell transferring of proteins, mRNAs, and miRNA, among others (68).

In this regard, exosomes have been proved to reflect stress conditions by modifying their protein or mRNA concentration (69). These molecular components associated with exosomes may play a role in a variety of cellular physiological mechanisms. Waldenström et al. have extracted total RNA from culture-isolated cardiomyocyte exosomes (cardiosomes) and have accomplished the identification of 1520 mRNAs similar to those found in cardiomyocytes. Moreover, almost one-third of these mRNAs had a direct relationship with biological mechanisms, including gene expression changes (70).

Generally, after myocardial infarction there is an alteration in circulating miRNAs which may act in cell–cell communication between cardiac cells and the bone marrow, opening up the possibility of generating a cardioprotective mechanism via the paracrine activation of cardioprotective kinase pathways (71). Chen et al. have demonstrated that exosomes derived from cardiac progenitor cells (CPCs) can protect cardiomyocytes from oxidative stress *in vitro* and *in vivo* by inhibiting ischemia/reperfusion-induced apoptosis, finding enrichment in miR-451 in CPC-derived exosomes when compared with CPC. The transcription of miR-451 responds to GATA4, a transcription factor associated with cardiac morphogenesis, cardiomyocytes survival, and cardiac function maintenance in the adult heart (72).

On the other hand, it has been proved that exosomes derived from cultured cells under hypoxia conditions contain fibronectin, collagen, and lysyl-oxidase-like 2 (LOXL2) in their protein content, suggesting the participation of these proteins in cytoskeletal and extracellular matrix rearrangements of neighboring or distant cells (69).

Moreover, Liao et al. have shown that cardiomyocytes are able to produce TNF-α after induction via hypoxia-inducible factor 1 (HIF-1). This cytokine is present in exosomes during hypoxic conditions and has been demonstrated to contribute actively to inflammation and cardiac remodeling (73).

All these features of exosomes have led Yellon and Davidson to name these nanovesicles “dark matter of the body” – invisible to direct microscopy, but whose existence can be inferred by the effects they have on other cells” (71).

Moreover, their capacity to transport such variety of molecules may allow exosomes to provide a biological “snapshot” of the cell physiological conditions (71) which, in turn, reflects the health status of the individual.

In summary, all these characteristics make exosomes dynamic vesicles endowed with the ability of transporting a great variety of molecules and, taking into account the specificity of their surface proteins, they have been promoted as specific therapeutic transporters for cardiovascular diseases.

## The Role of Exosomes in Pregnancy

To achieve a successful pregnancy, a specific suppression of the maternal immune system and a homeostatic balance to preserve an adequate utero-placental circulation are required. During this process various modulatory signals are released in different forms including EVs.

Pregnancy is an immunological phenomenon where the semiallogeneic fetus is not rejected because of the immune tolerance induced toward it. In this regard, exosomes with immunosuppressant activities were found to be increased in pregnant women, compared with non-pregnant ones. Moreover, exosomes isolated from sera of women with full-term pregnancies are present at significantly greater concentrations than those from pregnancies delivering pre-term.

The syncytiotrophoblast of the human placenta continuously and constitutively produces and secretes exosomes to the maternal bloodstream. These exosomes exhibit a redundant number of mechanisms that inhibit the function of the maternal immune system during pregnancy and promote the survival of the fetus. These microvesicles express significantly higher levels of the pro-apoptotic molecules Fas ligand (FasL), TRAIL, and PD-L1, inducing T-cell death. Placenta-derived exosomes which express FasL also suppress CD3-ζ chain and the enzyme Janus kinase 3 (JAK3), leading to T-cell anergy. In addition, it has been reported that placental exosomes also carry NKG2D ligand (the ligand molecule for the activating receptor NKG2D express on NK cells) that may downregulate the activity of NK cells by binding to NKG2D receptor and consequently impairing the maternal cytotoxic activity (74–76).

It has been reported that exosomes released in a primary culture of cytotrophoblast cells contain biologically active proteins that can interact with the maternal endothelium and regulate their function (e.g., migration and angiogenesis) (77). Furthermore, exosomes isolated from plasmas of pregnant healthy women in the first trimester promote vascular cell migration from the uterine spiral arteries and may play a role in regulating the endothelium response to maternal adaptation to pregnancy. However, under pathological conditions (e.g., proinflammatory states and pre-eclampsia), the bioactivity of placental exosomes is reduced (78).

Placental miRNAs are abundant in the plasma of pregnant women and are upregulated in pre-eclampsia. It has been proposed that these specific placental miRNA are extracellularly secreted to the maternal circulation from the syncytiotrophoblast through exosomes. The diagnostic and prognostic usefulness of exosomal miRNAs are presently being investigated. However, little is known about the role of placental exosome-associated miRNAs in maternal cells and tissues during pregnancy (79).

During pregnancy, maternal circulation is characterized for the presence of different EV simultaneously secreted by the syncytiotrophoblast, differing in size, morphology, and function (80). Syncytiotrophoblast-derived exosomes as stated earlier are immunosuppressive down-regulating maternal immune system. Syncytiotrophoblast-derived microvesicles/microparticles (STBM) are larger EV and may include apoptotic or necrotic material, the former immunosuppressive and the latter immunostimulatory, anti-angiogenic and procoagulant (80). It is suppose that during normal pregnancy, a delicate balance occurs between STBM and exosomes, favoring STMB according with a mild systemic inflammation distinctive of normal pregnancy. In contrast, in pathological condition such as infertility, recurrent abortions, pre-eclampsia, pre-term labor, and pre-mature birth, this balance is broken. For example, in pre-eclampsia, inflammatory stress could activate the syncytiotrophoblast causing it to release STMB with pro-inflammatory, anti-angiogenic, and procoagulant activities (80, 81).

Although the role of immunosuppressive placental exosomes during normal pregnancy is clear, the contribution of exosomes in pathological pregnancies and related diseases, such as recurrent abortions and infertility, need a more profound evaluation. The knowledge derived in these areas will open up possibilities for novel, exosome-based treatments of pregnancy failure and infertility.

## The Role of Exosomes in Infectious Diseases

Over the last years, it has been well documented that mammalian cells infected by single-cell eukaryotic/prokaryotic pathogens or even prions secrete exosomes with different purposes. Besides, bacteria secrete biological active vesicles named outer membrane vesicles (OMVs); fungi and eukaryotic parasites also produce EVs (82). Moreover, it is known that even parasitic trematodes and nematodes release exosomes as an immunomodulatory mechanism (83). Significantly, these microvesicles were demonstrated to play an important role in infection biology. Apart from the widely studied immunomodulatory effects, pathogen-released exosomes are known to carry specific virulence factors, such as proteins, mRNA, and miRNA, which contribute to spread the infection. As a consequence, microvescicles can either expand or contain the infection being thus beneficial for either the pathogen or the host.

Prions are abnormally folded proteins with the ability to propagate in the central nervous system causing fatal neurodegenerative disorders. Infectious prion proteins (PrPSc) have been identified in exosomes derived from the conditioned media of mammalian neurons (64, 66). These exosomes were internalized by bystander normal cells, transforming naturally occurring cellular prion proteins (PrPc) into misfolded infectious prion proteins (PrPSc), suggesting that exosomes may contribute to intercellular membrane exchange and dissemination of prions throughout the organism. Moreover, the intracerebral inoculation of exosomes obtained from infected cell cultures has proved to cause clinical disease in mice (64, 84). Recently, exosomes inclosing prion proteins were also isolated from plasma of mice bearing transmissible spongiform encephalopathy, suggesting a possible spread of transmissible spongiform encephalopathies via the blood (85).

In viral infections, microvescicles, and particularly exosomes, have been involved in various mechanisms, depending on the type of virus, its life cycle, and the type of infected cell.

HIV-1 virus has developed many exosome-mediated strategies to manipulate the host’s cell machinery. For example, exosomes take part in the transfer of proteins, and RNA (miRNA, sRNA) from infected to non-infected cells, transporting these components even to distant cells. Exosome-mediated transport and deliver of functional proteins to recipient cells has been demonstrated in HIV-1 infected macrophages (86). This phenomenon has been demonstrated for crucial proteins for HIV-1 infection such as Gag, p17, and Nef. For example, Nef is incorporated into exosomes released from infected cells and subsequently induces apoptosis of uninfected CD4+ T-cells contributing to viral immune suppression (87). In addition to the proteins, miRNAs transported by exosomes have been demonstrated to be involved in HIV-associated neuronal dysfunction and susceptibility to viral infection (88). In addition, HIV-1 particles captured by immature DCs (iDCs) are exocytosed in association with exosomes and could mediate trans-infection of CD4+ T-cells (89). On the other hand, HIV-1 has also developed a strategy known as *The Trojan horse hypothesis of HIV trans-infection* (90, 91). This model proposes that HIV-1 enters mature DC (mDC). The virus is retained in the MVB compartment and follows the same trafficking pathway used by DC exosomes for antigen dissemination to amplify antigen presentation during pathogen invasion (16, 91). Afterwards, mDC release them to trans-infect CD4+ cells, mainly in the lymph nodes (90–92).

Another retrovirus infection in which exosomes play an important role during pathogenic viral infection is the Human T-lymphotropic virus type 1 (HTLV-1). The extracellular delivery of functional HTLV-1 proteins as the trans-activator protein Tax, as well as viral mRNA transcripts including Tax, HBZ, and Env to uninfected recipient cells via exosomes, protect them from apoptosis under stress conditions and transfers functional HTLV-1 molecules to this uninfected recipient cells contributing to the pathogenesis of HTLV-1 (93).

Recent evidence indicates that Hepatitis B, C, and E viruses (HBV, HCV, and HEV) also employ the exosomal pathway machinery to mediate alternative active viral transmission and disease persistence. Moreover, it has been demonstrated that exosomes isolated from sera of chronic HCV-infected patients contain HCV RNA, and these exosomes could mediate viral receptor-independent transmission of HCV to uninfected hepatocytes (94). Furthermore, the exosomal export of viral RNA may serve both as a viral strategy to evade the immune system spreading the infection, and as a host’s strategy to induce an innate response in bystander cells. In this regard, exosomes derived from HCV-infected cells can also induce the production of type I IFN by the transference of immunostimulatory viral RNA from infected cells to DCs and trigger the production of IFN-α (95).

Members of the Herpes virus family, such as Human Herpes Virus and Herpes simplex, Epstein–Barr virus and human Cytomegalovirus, have also been studied for their interaction with cellular exosomes. As described for retroviruses, the Herpes virus captures the cellular pathway for exosomes biogenesis and release. In addition, enhances virus loaded exosomes production, supporting the persistence and dissemination of viral particles. Furthermore, exosomes may contribute either to the viral escape from the immunological surveillance or to interfere with the transport of antigens to the host’s immune system (96).

Exosomes derived from Epstein–Barr virus-infected cells have also been involved in MVB biogenesis, viral egress, and infection to neighboring uninfected cells. In addition, Epstein–Barr virus-infected B-cells can deliver viral BART miRNA to DCs, thus leading to the exosomal-dependent immune suppression in EBV-associated lymphomas (97, 98).

Gram-negative bacteria release OMV to communicate with prokaryotic and eukaryotic cells. They carry and transmit virulence factors, mediate bacterial binding and invasion, cause cytotoxicity, and modulate the host’s immune response (99, 100). Furthermore, exosomes secreted by human infected host cells play a relevant role in the bacteria–host interaction. This characteristic is especially significant to identify intracellular pathogens, as bacterial products are not always readily present in fluids for the pathogen detection and diagnosis. In this regard, the works done with *Mycoplasma tuberculosis* and *M. bovis* have yielded relevant results. *M. tuberculosis* or *M. bovis*-infected macrophages release exosomes containing pathogen-derived antigens and these vesicles activate both the innate and acquired immune responses (46, 101, 102) inducing the production of pro-inflammatory cytokines by naïve cells. Exosomes expressing mycobacterial molecules have been detected in the serum of patients with acute and latent tuberculosis infection. These findings not only enabled the diagnosis but also served as active or latent phase markers of the disease (103).

The expression of specific bacterial proteins either on OM or on exosomes derived from infected as well as from DCs pulsed with microorganism peptides opened up a new alternative for the development of prophylactic or therapeutic bacterial cell-free vaccines.

Finally, exosomes from the fungus *Cryptococcus neoformans* and from parasites such as *Trichomonas vaginalis*, *Trypanosoma cruzi*, *Leishmania* spp., *Plasmodium falciparum, Toxoplasma gondii* (104), and helminthes (83) have also been described. These vesicles express virulence factors and, in addition, stimulate both pro-and anti-inflammatory responses in host cells (104). Interestingly, Regev-Rudzki et al. have reported *P. falciparum* within red blood cells directly communicate to the parasites population using exosome-like vesicles that are capable of delivering genes (105), inducing the promotion and differentiation to sexual form of the parasite.

As stated earlier, the exosomes secreted in response to an infection are implicated in many processes of the infection biology. The exosomes isolated from infected cells may be regulated to eliminate or to attenuate virulence factors to control the spreading of the infection. As they modulate the immune response, exosomes derived from the host’s cell or from pathogens could be novel candidates for the design of acellular vaccines. In addition, exosomes can also be employed as infection biomarkers.

## Diagnosis and Potential Clinical Application of Exosomes

The aim of this review was to discuss some significant aspects of exosomes. First, exosomes may be isolated from almost every cell, not only from eukaryotic but also from prokaryotic cells. Second, microvesicles participate in the regulation of central normal biological processes such as the immune response, pregnancy, tissue repair, and blood coagulation, among others. Third, exosomes are involved in pathobiological mechanisms related to the most frequent types of diseases affecting the population such as neurodegenerative disorders, tumorigenesis, and infectious diseases. Fourth, these microvesicles may be captured specifically by cells where they can deliver their content, and finally, they are very stable both *in vivo* (in systemic circulation) and *in vitro* (they can be preserved frozen for a long period of time without losing their biological properties) (15).

**Figure 1:**
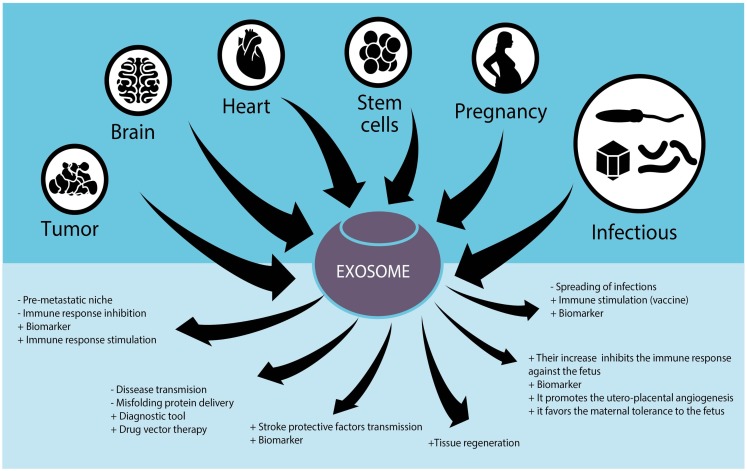
**Role of exosomes in different tissues and their potential use in human diseases**.

Given the importance of exosomes in normal and pathobiological conditions, microvesicles are being studied for their potential therapeutic uses. For example, they are evaluated as biomarkers for the diagnosis and disease follow-up, as immunomodulators to suppress or stimulate the immune system, as vectors for drug delivery, and as therapeutic agents *per se* (Figure 1).

One of the hallmark properties of exosomes is the expression of specific proteins or miRNA belonging to the cells from which they derive. In addition, they may be isolated from fluids such as blood, urine, saliva, amniotic fluid, malignant ascites, bronchoalveolar lavage fluid, synovial fluid, and breast milk. These characteristics were the starting point of many of studies aiming at proving exosomes as potential biomarkers for the diagnosis and prognosis of diseases.

Nowadays, cancer diagnostics rely on biopsies. The potential uses of exosomes have the advantage of being a sensitive and non-invasive method, allowing the detection of tumors at an early stage. Cancer exosomes have been found to be useful for the detection of many types of tumors: prostate, breast, and ovarian cancer; glioblastoma and melanoma, among others (106–110). As mentioned earlier, exosomes transport misfolded proteins associated to neurodegenerative disorders. In Alzheimer’s disease, the detection of α-amyloid 42 and tau proteins was possible in cerebrospinal fluid samples at an early stage of the disease, thus opening up the possibility of an early detection (111, 112). A number of works have described the association of exosomes isolated from urine with several kidney pathologies such as renal ischemia/reperfusion, nephrotic syndrome, and acute kidney injury (113–115). The levels of circulating placental exosomes expressing immunosuppressive molecules (FasL) are under investigation to be used as a biomarker to detect complicated pregnancies (75).

Finally, exosomes proved to be a good marker for the diagnosis of infectious diseases. Infectious RNA and proteins are expressed on exosomes, including viral and misfolded proteins allowing the development of sensitive tests, not only to detect the causative agent but also to followup the infection. In this regard, exosomes are especially useful to detect active and latent forms of intracellular infections, such as tuberculosis. In a recent study, 33 unique proteins of *Mycobacterium tuberculosis* were identified from exosomes isolated from human serum that may serve as biomarkers for persistent active and latent tuberculosis (103). Exosomes from human serum are also employed to detect HIV-positive patients and this test is at present commercialized (116).

Another field of intensive study is the use of exosomes to modulate the immune system. Because the pioneer works of Raposo et al. demonstrated that exosomes derived from antigen-presenting cells (B-cells and DCs) contained both MHC I and MHC II molecules and that they could elicit a specific anti-tumoral immune response (22), a myriad of studies employing exosomes as acellular vaccines have been published not only for cancer but also for infectious diseases. As already stated, numerous Phase I protocols have been conducted in cancer patients using an exosomal vaccination. Among them are the vaccination of metastatic melanoma patients (5), advanced non-small cell lung cancer with autologous DC-derived exosomes (117), and patients with colorectal cancer immunized with tumor exosomes derived from ascitic fluid (118).

Following the same strategy, protocols employing DCs pulsed with peptides obtained from infectious agents were developed. For instance, DC pulsed with diphtheria toxin (119), or with *Streptococcus pneumoniae* capsular polysaccharide Cps14 (120), for bacterial diseases and with sonicates from tachyzoites of *Toxoplasma gondii* (47) were investigated.

As immunomodulators, exosomes may be either immunoactivating or immunosuppressive agents. Exosomes were proposed as acellular antigens for the development of vaccines against either infectious diseases or tumors. For example, immunization with exosomes derived from reticulocytes infected with a *Plasmodium yoelli* elicits protective immune responses. This strategy offers a novel platform to develop a vaccine against malaria infection to induce full- and long-lasting protection on this lethal infection (121). Cancer vaccines employing unmodified exosomes (24, 122) or exosomes engineering to express particular molecules derived from tumor cells such as IL-18 or HSP (123–125) induced specific anti-tumor immunity. Using immunosuppressive exosomes from iDCs, it has been possible to induce a donor-specific suppression in a mismatched cardiac allograft model (126, 127). EVs derived from genetically modified DCs expressing FasL, IL-10, or IL-4 successfully suppressed delayed-type hypersensitivity in a model of collagen-induced arthritis (43, 44, 128, 129).

Exosomes are also important therapeutic agents *per se*. EV derived from mesenchymal stem cells have the ability to induce tissue regeneration by delivering growth factors, proteins, miRNA, mRNA non-coding RNA, and lipids. Several reports have demonstrated the feasibility to regenerate cardiac tissue and neo-vascularization in models of myocardial infarction and kidney injury using exosomes from steam cells (MSC-exo) and endothelial progenitor cells (130). A recent paper by Kordelas et al. (131) has reported a successful therapy for refractory graft-versus-host disease (GvHD) employing MSC-exo in a pediatric patient. In this regard, MSC-exo are under approval for pediatric GvHD treatment in Canada and New Zealand (132). In remarkable studies, it has been demonstrated that systemic administration of MSC-exo effectively improved functional recovery in rats and after stroke (133), and after traumatic brain injury (134) by increasing neurite remodeling, neurogenesis, and angiogenesis.

Owing to their natural origin, exosomes constitute an efficient tissue-specific, non-immunogenic carrier to deliver therapeutic drugs. The fact that exosomes can be engineered to express foreign proteins, miRNA and also siRNA, allows not only the expression of proteins in a tissue-specific manner, but also the silencing of specific genes. For instance, exosomes effectively delivered siRNA can knockout genes inside tumor target cells *in vitro* (135) and *in vivo* into neuronal cells by intravenous injection of the modified vesicles (63). Similarly, mRNA was efficiently incorporated to and expressed in tumor cells (136). These approaches illustrate the possibility to use human exosomes as vectors in RNA-based gene therapy for neurodegenerative disorders, cancer as well as for the treatment of other pathologies.

As the potential therapeutic application of exosomes has gained considerable interest, an important point is starting to be considered. It refers to the kinetics, biodistribution, and clearance of exosomes on systemic administration. Recent studies alert about the rapid clearance of exosomes by the liver and spleen (137–139). In a recent work by Smyth et al., they observed a rapid clearance and minimal tumor accumulation of intravenously injected tumor exosomes, limiting their employment as drug delivery vehicle. However, a significant greater concentration of the same exosomes were retained in the tumor when delivered intratumorally (137). To overcome these problems Rana et al. demonstrated that exosomes may be selectively taken up by target cell by altering the expression of different tetraspanin proteins (140).

Further studies are needed to overcome these challenges and to establish if exosomes could be utilized effectively for drug delivery.

## Concluding Remarks

The interest in exosomes is mainly because of the important biological and pathobiological functions in which they are involved. In addition, they offer multiple new therapeutic possibilities because of their properties that are used to detect and to ameliorate or cure severe diseases.

Regardless the potential benefits to employ exosomes for therapeutics that were described, some issues such as the purity of the exosomes preparation, the co-expression of different molecules within the microvesicle (proteins and RNAs that may interfere in the biological function required), and the administration route to achieve the targeted delivery and the desired effect remain to be solved. Future research will improve the methods for isolation of pure exosomes necessary to distinguish them from other types of microvesicles and to understand the mechanism in which they are involved. These achievements will help bolster the early diagnosis, control, prevention and therapy.

## Conflict of Interest Statement

The authors declare that the research was conducted in the absence of any commercial or financial relationships that could be construed as a potential conflict of interest.
